# Microparticle-mediated VZV propagation and endothelial activation: Mechanism of VZV vasculopathy

**DOI:** 10.1212/WNL.0000000000008885

**Published:** 2020-02-04

**Authors:** Despina Eleftheriou, Elena Moraitis, Ying Hong, Mark Turmaine, Cristina Venturini, Vijeya Ganesan, Judith Breuer, Nigel Klein, Paul Brogan

**Affiliations:** From the Infection, Immunology and Rheumatology Section (D.E., E.M., Y.H., C.V., J.B., N.K., P.B.) and Clinical Neurosciences (V.G.), University College London GOS Institute of Child Health; Arthritis Research UK Centre for Adolescent Rheumatology (D.E.); and Department of Cell and Developmental Biology (M.T.), University College London, UK.

## Abstract

**Objective:**

Varicella zoster virus (VZV) can spread anterogradely and infect cerebral arteries causing VZV vasculopathy and arterial ischemic stroke. In this study, we tested the hypothesis that virus-infected cerebrovascular fibroblasts undergo phenotypic changes that promote vascular remodeling and facilitate virus transmission in an in vitro model of VZV vasculopathy. The aims of this project were therefore to examine the changes that virus-infected human brain adventitial vascular fibroblasts (HBVAFs) undergo in an in vitro model of VZV vasculopathy and to identify disease biomarkers relating to VZV-related vasculopathy.

**Methods:**

HBVAFs were infected with VZV, and their ability to migrate, proliferate, transdifferentiate, and interact with endothelial cells was studied with flow cytometry. Microparticles (MPs) released from these cells were isolated and imaged with transmission electron microscopy, and their protein content was analyzed with mass spectrometry. Circulating MP profiles were also studied in children with VZV and non-VZV vasculopathy and compared with controls.

**Results:**

VZV-infected HBVAFs transdifferentiated into myofibroblasts with enhanced proliferative and migratory capacity. Interaction of VZV-infected HBVAFs with endothelial cells resulted in endothelial dysfunction. These effects were, in part, mediated by the release of MPs from VZV-infected HBVAFs. These MPs contained VZV virions that could transmit VZV to neighboring cells, highlighting a novel model of VZV cell-to-cell viral dissemination. MPs positive for VZV were significantly higher in children with VZV-related vasculopathy compared to children with non-VZV vasculopathy (*p* = 0.01) and controls (*p* = 0.007).

**Conclusions:**

VZV-infected HBVAFs promote vascular remodeling and facilitate virus transmission. These effects were mediated by the release of apoptotic MPs that could transmit VZV infection to neighboring cells through a Trojan horse means of productive viral infection. VZV+ MPs may represent a disease biomarker worthy of further study.


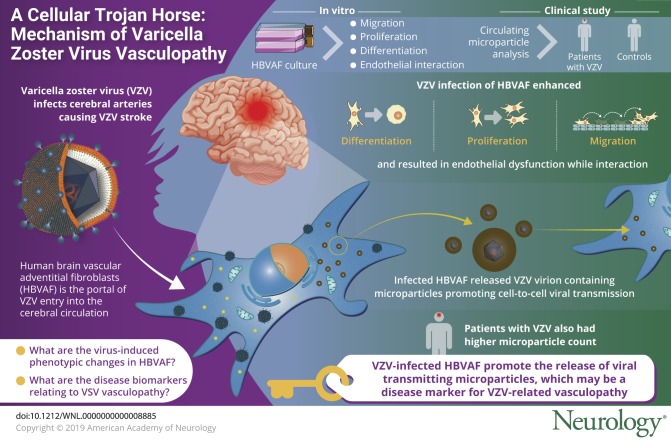


Varicella zoster virus (VZV) can spread anterogradely and infect cerebral arteries, causing VZV stroke.^1^ Recent histologic studies from VZV-infected brain arteries indicate that human brain vascular adventitial fibroblasts (HBVAFs) are the portal of VZV entry into the cerebral circulation and may be the source of myofibroblasts identified in the intima of VZV-infected cerebral arteries.^1^ We examined the changes that these virus-infected cerebrovascular fibroblasts undergo to promote vascular remodeling and facilitate virus transmission in an in vitro model of VZV vasculopathy. We identified that these effects were, in part, mediated by microparticles (MPs) containing virions that transmitted VZV to neighboring cells.

## Methods

### VZV culture and viral protein detection

THA- or green fluorescent protein (GFP)-tagged ORF 23-expressing VZV was grown in MeWo (human melanoma cell line) cells, mitotically inactivated by mitomycin C, and titered by immunohistochemical staining with anti-VZV antibodies. Infected HBVAFs (either VZV or mock) were inoculated to resting HBVAFs at a ratio of 1:3. Infection was confirmed with detection of cytopathic effect and expression of the following viral proteins: envelope glycoprotein H (gH) expressed at 12 to 24 hours after VZV infection and VZV immediate early protein (IE62 protein) expressed at 4 to 12 hours after VZV infection.^2^

HBVAF transdifferentiation and proliferation were assessed with α-smooth muscle actin–APC (R&D Systems, Minneapolis, MN) and Click-iTTM Plus EdU Alexa Fluor 647 Kit (Invitrogen, Carlsbad, CA). HBVAF migration was assessed using a scratch assay with a 200-μL pipette tip in cells incubated with 10 μg/mL mitomycin C to inhibit mitosis.

CD54-PE (BD Biosciences, East Rutherford, NJ ) expression on human umbilical vein endothelial cells (HUVECs [PromoCell, Heidelberg, Germany]) was analyzed by flow cytometry. Intracellular reactive oxygen species (ROS) production was determined as oxidation-dependent fluorescence of dichloro-dihydro-fluorescein diacetate (Molecular Probes, Eugene, OR).

Cytokines were measured with Meso Scale Discovery electrochemilluminescence.

MPs were sedimented from 200 μL platelet-poor plasma or culture supernatants after centrifugation at 17,000*g* for 60 minutes and stained with phycoerythrin-labeled annexin V (BD Biosciences) or mouse anti-gH VZV monoclonal antibody (Abcam, Cambridge, UK).

Negative-stain transmission electron microscopy was performed on MPs obtained by sequential centrifugation at 131,000*g* for 1 hour. Samples were loaded onto a copper carbon/formvar-coated grid, negatively stained with 2% aqueous uranyl acetate, and imaged with a JEOL JEM-1010 electron microscope.

For proteomics, solid-phase extraction was performed before liquid chromatography/mass spectroscopy analysis. Samples were analyzed on nanoelectrospray IMS QToF (Synapt 2GSi). Proteins with a Protein Lynx Global Server score >95% in the Uniprot Human Proteome database were considered.

### Patients

We recruited patients with cerebral vasculopathy (n = 3 with VZV vasculopathy, n = 10 with non-VZV vasculopathy) (table) referred to the neurovascular service at the Great Ormond Street Hospital NHS Foundation Trust, London, UK. Arterial ischemic stroke (AIS) was defined as an acute focal neurologic deficit attributable to cerebral infarction in a corresponding arterial distribution.^3^ Cerebral/cervical vasculopathy/arteriopathy was defined as focal or segmental stenosis or occlusion with abnormalities of the arterial wall and categorized according to current consensus definitions.^3^ For the 10 patients with non-VZV–related cerebral vasculopathy, the following tests were negative or normal: intrathecal VZV immunoglobulin (Ig) G production, CSF PCR for VZV, and blood test investigations (serum IgM and VZV PCR) for acute VZV infection. There was no history of chickenpox in any of these 10 children. Three patients were classified as having VZV vasculopathy on the basis of radiologic evidence of cerebral vasculopathy/arteriopathy and history of VZV infection within 12 months from the acute AIS event and/or positive CSF (VZV PCR or intrathecal VZV IgG) findings.^4^ Age-matched healthy controls were children with no AIS, no acute or recent history of VZV infection, or any other vascular pathology (n = 10).

**Table:**
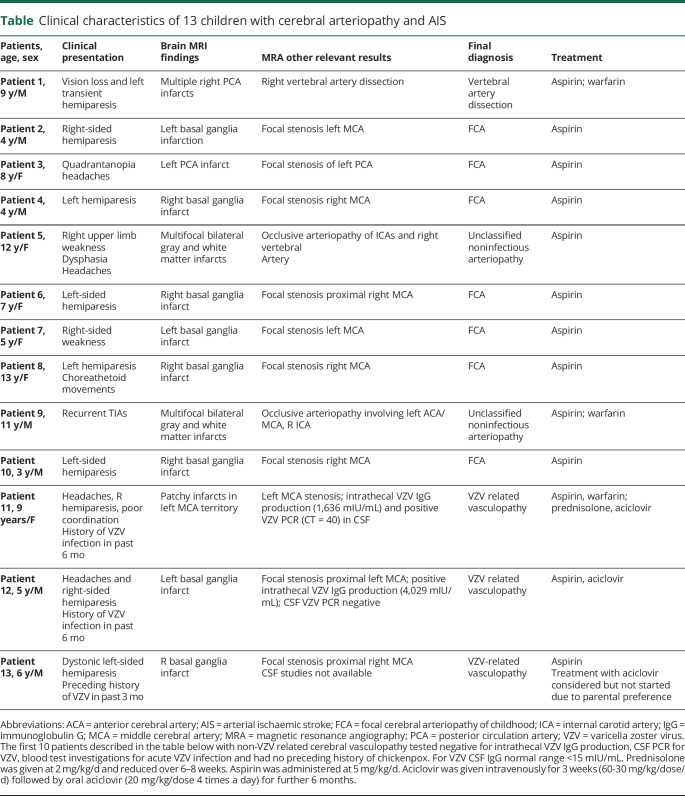
Clinical characteristics of 13 children with cerebral arteriopathy and AIS

### Standard protocol approvals, registrations, and patient consents

This study was approved by the Bloomsbury Ethics Committee, London (07/Q0508/58), and all participants provided consent or age-appropriate assent.

### Statistics

Results are expressed as mean and SEM or median and range. Values of *p* < 0.05 by unpaired *t* test or Mann-Whitney *U* test were considered significant.

### Data availability

All data are presented within this article and its supplemental material.

## Results

First, we confirmed that VZV could infect HBVAFs. Cells were infected/mock infected with either wild-type VZV or GFP-ORF23 VZV. We detected positive expression of GFP-ORF23 and of VZV-related viral proteins gH and VZV IE62 in infected HBVAFs, with no detectable VZV protein or GFP-ORF23 expression in mock-infected cells from day 2 onward.

We observed increased myofibroblast transdifferentiation of VZV-infected HBVAFs compared to mock-infected cells, as indicated by increased expression of α-smooth muscle actin (a myofibroblast surface marker) in VZV-infected HBVAFs at 2 and 6 days after infection (*p* = 0.04 and *p* = 0.03, respectively). VZV infection also enhanced proliferation of HBVAFs, as indicated by increased expression of the marker EdU in VZV-infected cells, compared to mock-infected cells at days 2 and 6 (*p* = 0.03 and *p* = 0.01, respectively). Significantly more HBVAFs migrated into the marked scratch area of an in vitro scratch assay at 17 hours in the VZV-infected cultures compared to mock-infected cultures (*p* = 0.01).

Adventitial fibroblasts produce biologically active proteins that affect endothelial function and vasculopathy progression.^5^ Therefore, we next examined the interaction of VZV-infected HBVAFs with endothelial cells. Conditioned media from VZV-infected HBVAFs induced significant upregulation of CD54 expression, a marker of endothelial activation, in cultured HUVECs (*p* = 0.0009) and resulted in enhanced ROS production as assessed through oxidation-dependent fluorescence of dichloro-dihydro-fluorescein (*p* = 0.0005). No such changes were observed with media from mock-infected cells.

To further characterize what could be driving this endothelial activation, we profiled cytokine production in the culture supernatant from VZV-infected HBVAF compared to mock-infected cells. These cells released significantly higher levels of cytokines/chemokines that activate endothelium, promote leucocyte adhesion, and enhance fibroblast migration: interleukin-6 (*p* = 0.02); tumor necrosis factor-α (*p* = 0.02); interleukin-8 (*p* = 0.018), and monocyte chemoattractant protein-1 (*p* = 0.02).^5^

We next investigated signaling between HBVAFs and endothelial cells mediated by cellular MPs. MPs (0.1–1 μm) are released from apoptotic or activated cells and have high surface phosphatidylserine content, which binds annexin V.^3^ We identified a significantly higher number of annexin V+ GFP+ ORF23+ MPs in supernatants from cultures of VZV-infected cells compared to mock-infected cells (*p* = 0.02). These MPs also expressed the VZV protein gH.

Transmission electron microscopy uranyl acetate-negative staining of MPs confirmed the presence of VZV associated with MPs in pellets from VZV-infected cells; these virus particles were not detected in MP pellets from mock-infected cells (figure, A–D). MPs were identified in the VZV-infected pellets with entrapment of viral particles inside MP membrane coats. Viral particles were discernible in the MP structures and were morphologically distinct with an average size of 80 nm. In contrast, MPs had a size of 100 to 400 nm (figure, D).

**Figure:**
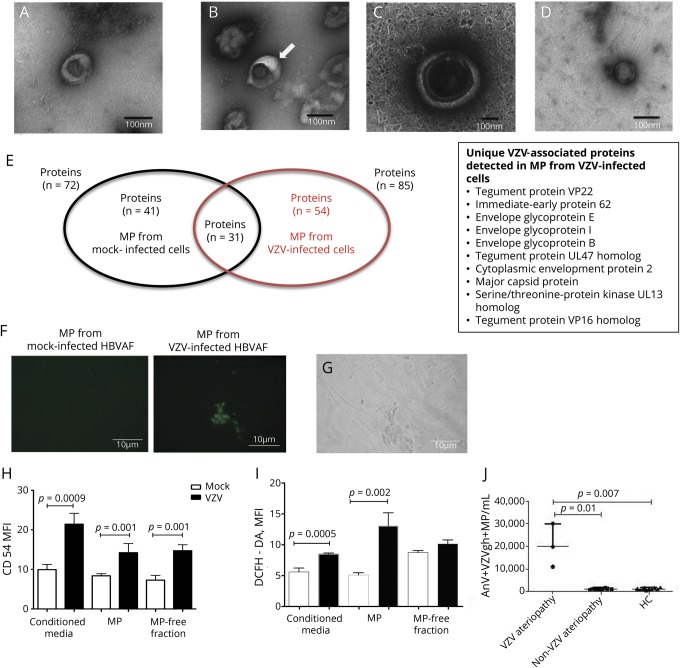
VZV induces release of MPs that contain VZV particles (A–D) Transmission electron microscopy images of microparticle (MP) pellet obtained at 135,000×  and 95,000× magnification. Scale bar 100 nm. (A) MP shed by mock-infected human brain adventitial vascular fibroblast (HBVAF) that appears collapsed and donut shaped (diameter 100 nm). (B) MP (arrow) harvested from varicella zoster virus (VZV)-infected HBVAF closely associated with a viral particle. (C) MP with a diameter of 400 nm includes a viral envelope as a central spherical structure of ≈80-nm diameter with surface spikes. (D) Free VZV particle with 80-nm diameter, penetrated by the stain, with the appearance of a hexagon with surface projections (viral nucleocapsid). No envelope was identified outlining the nucleocapsid. (E) Proteins identified by liquid chromatography-mass spectrometry in MP from control mock-infected HBVAF and from VZV-infected HBVAF. (F and G) MP induced VZV infection in cultures of HBVAF shown with both phase-contrast and fluorescent microscopy and with no such changes in control. (H and I) Incubation of endothelial cells with MP induced upregulation of CD54 expression (*p* = 0.001) and reactive oxygen species, determined as oxidation-dependent fluorescence of dichloro-dihydro-fluorescein diacetate (DCFH-DA, *p* = 0.0002). Results are expressed as mean and SEM; *p* values were calculated by the unpaired *t* test. (K) Higher numbers of AnV+ VZV- glycoprotein H+ MPs were detected in the circulation of children with VZV-related vasculopathy compared to children with other vasculopathies (*p* = 0.01) and controls (*p* = 0.007). Results are expressed as median and range; *p* values were calculated by Mann-Whitney *U* test. AnV = Annexin V; MFI = mean fluorescent intensity.

Proteomic analysis of MPs derived from VZV-infected HBVAFs revealed proteins consistent with their fibroblastic origin. MPs were highly enriched for VZV proteins that were not detected in the MPs derived from mock-infected samples (figure, E and data available from Dryad, doi.org/10.5061/dryad.466fk4h) and did not express RAB (Ras superfamily of monomeric G) proteins commonly found in endosomes.^6^

We next tested whether MPs were able to induce productive infection. Cytopathic effect was observed in HBVAFs incubated with MPs derived from VZV-infected cells using both phase-contrast and fluorescent microscopy, with detection of green fluorescence representing GFP-ORF23 VZV. No fluorescence was detected in HBVAFs incubated with MPs from mock-infected HBVAFs (figure, F and G). The addition of heparin, an inhibitor of cell free virus entry to the cells, made no difference in the infection efficiency observed, providing evidence that the infection was mediated by MP-VZV complexes, not free VZV. MPs from infected HBVAFs were also able to activate endothelium, as demonstrated by higher induction of CD54 expression (*p* = 0.001) and higher ROS production (*p* = 0.002) in HUVECs compared to MPs from mock-infected HBVAF (figure, H and I).

Lastly, we explored VZV-MP complexes in the circulation of children with AIS and VZV-related cerebral vasculopathy, children with cerebral vasculopathy not associated with antecedent VZV, and healthy age-matched controls. Total annexin V+ MP levels were higher in children with VZV vasculopathy (n = 3) compared to healthy controls (n = 10, *p* = 0.007) but did not differ from total annexin V+ MP levels detected in patients with non-VZV–related cerebral vasculopathy (n = 10). When costained for the detection of VZV gH protein, however, a significantly higher number of annexin V+ VZVgH+ MPs were identified in children with VZV-related vasculopathy compared to children with non-VZV vasculopathies (*p* = 0.01) and controls (*p* = 0.007, figure, K).

## Discussion

Our findings support a novel model of VZV vasculopathy pathogenesis, with adventitial fibroblast transformation to myofibroblasts contributing to arterial remodeling by proliferation and migration and further adventitial cell-to-cell viral dissemination and endothelial activation mediated by VZV-MPs. MPs are important intercellular signaling mediators,^3,7^ but few studies have demonstrated their role as mediators of infectious propagation.^8^ Viral proteins detected in MPs could facilitate interaction on host cells and may also regulate tropism. Virion transport by MPs may also represent a Trojan horse means of productive viral infection, with MP facilitating virus propagation while evading immune detection. MPs may deliver antigens derived from the biological cargo acquired from their cells of origin to antigen-presenting cells to abrogate early immune responses to viral invasion. This may not apply just to MPs; alternative cellular microvesicle populations (such as endosomes) may also play a role. Moreover, whether MP-mediated viral propagation is specific to VZV or also applies to other viruses causing AIS requires further investigation. It would also be interesting to establish whether VZV-infected cells release phenotypically and quantitatively distinct MPs in resting, activated, and apoptotic states.

The small size of our clinical study with a few cases with VZV vasculopathy precludes any definitive conclusions about our proposed mechanism in patients but does at least provide some preliminary face validity regarding our model in a clinical context for this rare disease. Although applied to a pediatric cohort here, the detection of MP-VZV complexes might also be an approach that is diagnostically useful for adult patients, in whom establishing a diagnosis of VZV vasculopathy can be equally challenging. Thus, while the diagnostic or prognostic relevance of circulating VZV+ MPs is currently unknown, study of this as a potential novel biomarker could be fruitful. This would require a prospective study of large numbers of patients, with or without vasculopathy, and further characterization of the cellular origin of these circulating MP-VZV complexes. It would also be interesting to use sensitive assays such as fluorescent antibody against membrane antigen and VZV-IgG time-resolved fluorescence immunoassay to quantify varicella antibodies in patients with VZV vasculopathy and correlate them with levels of VZV MPs.

While challenging in a rare disease setting in which patient numbers are limited, further insights might be obtained by exploring our model in more complex 3-D in vitro tissue culture systems. This might unveil novel druggable targets for VZV vasculopathy.

## Glossary

AIS: arterial ischemic stroke
GFP: green fluorescent protein
gH: glycoprotein H
HBVAF: human brain vascular adventitial fibroblast
HUVEC: human umbilical vein endothelial cell
Ig: immunoglobulin
MP: microparticle
ROS: reactive oxygen species
VZV: varicella zoster virus
